# The value of large-scale programmes in human genomics

**DOI:** 10.1038/s41431-025-01844-7

**Published:** 2025-04-08

**Authors:** Ruth Horn, Angeliki Kerasidou, Jennifer Merchant, The UK-FR+GENE (Genetics and Ethics Network) Consortium, Mark Bale, Natalie Banner, James Buchanan, Anne Cambon-Thomsen, Herve Chneiweiss, Lene Cividanes, Angus Clarke, Janneke M. L. Kuiper, Celine Lewis, Anneke Lucassen, Elizabeth Ormondroyd, Michael Parker, Eva Winkler

**Affiliations:** 1 Ethics of Medicine, Institute for Ethics and History of Health in Society, Faculty of Medicine, University of Augsburg, Augsburg, Germany; 2 The Ethox Centre, Nuffield Department of Population Health, University of Oxford, Oxford, UK; 3 CNRS Law&Humanities/CERSA UMR-7109, University Paris-Panthéon-Assas, Paris, France; 4 Institut Universitaire de France, Paris, France; 5 PHG Foundation, London, UK; 6 Genomics England, London, UK; 7 Health Economics and Policy Research Unit and NIHR Barts Biomedical Research Centre, Queen Mary University of London, London, UK; 8 Inserm, University of Toulouse III, Toulouse, France; 9 CNRS, Inserm, Sorbonne University, Paris, France; 10 National Genome Center, København, Denmark; 11 School of Medicine, Cardiff University, Cardiff, UK; 12 Life Sciences and Society Lab, KU Leuven, Leuven, Belgium; 13 Population, Policy and Practice, University College London, London, UK; 14 Nuffield Department of Medicine, University of Oxford, Oxford, UK; 15 Oxford NIHR Biomedical Research Centre and Department of Vascular Medicine, University of Oxford, Oxford, UK; 16 NCT Heidelberg, University of Heidelberg, Heidelberg, Germany

**Keywords:** Health policy, Genetics research

## Abstract

Large national genomic programmes have been created in many countries, including France, England and Germany, to advance the realisation of the potential genomic medicine holds to significantly contribute to society by improving health, and driving science, innovation and the economy. To reach this ambition, these programmes collect, manage and analyse big genomic datasets. While there is much talk about the promises, and hence the importance of genomics, there is little in-depth analysis of the actual contribution or value—here understood as benefits—of genomics for society at large. To explore the issue of the value of large-scale genomic programmes for society, UK-FR-D+ GENE held an international workshop focusing on a variety of levels—societal, economic, clinical, scientific, and population-wide level—at which such benefits might be observed. First, the broader societal implications of large genome programmes and their impact for public trust were discussed. Second, the meaning of fair and just allocation of public resources, based on considerations of the economic costs and benefits of genomic innovations, was examined. Third, the benefits of these innovations for stakeholders (clinicians, patients, and families) at the clinical level were investigated. Fourth, the scope and limitations of genomics at the scientific level were discussed. Finally, the potential of genomics to improve health at the population level was explored. Providing an insight into the benefits of large genomic programmes on various levels, the workshop concluded by defining several criteria that should be considered to ensure benefits for society when implementing large genomic programmes.

## Introduction

Genomic medicine has the potential to make significant contributions to society such as improving health, and driving science, innovation and the economy. To advance the realisation of this potential, large national genomic programmes have been created in many countries, including France, England and Germany. These programmes collect, manage and analyse big genomic datasets to reach this ambition.

While there is much talk about the promises and hence the importance of genomics, there is little in-depth analysis of the actual contribution or value of genomics for society at large. This question is of particular importance where national genomic programmes such as Genomics England, the Plan France Médecine Génomique 2025, or the German genomeDE are publicly funded and rely on citizens’ data being collected through public healthcare systems. As concluded in the last Consortium paper of the UK-France-Germany Genomics and Ethics Network (UK-FR-D+ GENE) [1], as well as by other authors, an important requirement for justifying the use of public data and resources to develop innovative health technologies within a publicly funded solidarity-based health system, is the equitable return of benefits of these technologies to society as a whole [2].

To explore the issue of the value of large-scale genomic programmes for society, UK-FR-D+ GENE held its fourth workshop on June 17th–June 18th 2024, inviting ethicists, social scientists, geneticists, clinicians and policy-makers from the UK, France, Germany, Belgium, Denmark, the Netherlands, Poland, and the European Commission to share their insights on this topic. Value here is understood as the practical worth—the tangible benefits—of large genomic programmes for society, and not as moral values and norms, which were discussed by the network previously [3]. Acknowledging the difficulty of determining these benefits, the workshop focused on a variety of levels—societal, economic, clinical, scientific, and population-wide level—at which such benefits might be observed. First, it was considered important to critically discuss the broader societal implications of large genome programmes and their impact for public trust. Second, the particiants examined what fair and just allocation of public resources might mean, based on balancing the costs and benefits of genomic innovations at the economic level. Third, it was investigated the benefits of these innovations for stakeholders (clinicians, patients, and families) at the clinical level—for example the way in which advancements in genomic medicine might impact the work of clinicians who practice it by providing them with new tools to help them offer better care to their patients, and of patients who receive genomic medicine. Fourth, the participants discussed the scope and limitations of genomics at the scientific level. Finally, the workshop focused on the question of the potential of genomics to improve health at the population level.

In essence, the workshop proposed a taxonomy of the benefits of large genomic programmes on a societal, economic, clinical, scientific, and population-wide level by drawing on insights from and discussions with a range of stakeholders. A number of criteria emerged from the discussions that most likely should be taken into account to ensure benefits for society when implementing large genomic programmes. These criteria are presented in the discussion of this manuscript.

## Societal level

Large-scale genomic programmes are being developed to enable better health, not just for the individual, but for society at large. For example, genomics might offer new ways of distinguishing individuals and sub-groups within populations, which in turn can take public health beyond the traditional parameters of disease risk such as age, sex, and socio-economic status. It might also enable the stratification and subsequent screening of individuals and population sub-groups based on their level of genetic risk for developing a disease. Meanwhile, the potential to develop targeted preventive solutions to reduce the burden of disease is appealing.

Whether these potential benefits can be converted into actual social benefits depends, among other factors, on whether people participate in this endeavour by donating their data, accessing available infrastructures and getting involved in research. To do this, people need to first trust the endeavour. Indeed, initial discussions raised the issue of public trust regarding the implementation of large-scale genomic programmes. As established in previous network workshops, public trust is central for the successful development of genomic medicine relying on the collection of large datasets [4]. However, public trust can also be put to the test by large data collection and sharing programmes that raise concerns about confidentiality and privacy issues [5]. This is particularly so where private companies gain access to data that was collected within the public healthcare system, and it is not clear how the data will be processed by these companies, or whether these partnerships aim to promote public benefit [1, 6]. As a response to this, democratic welfare states in Europe, and elsewhere, have made an effort to put in place strategies to gain and maintain support from a broad variety of different groups within society through rigorous data governance or public engagement and/or involvement programmes [4, 7].

In our workshop, it was argued that while it is important to value trust, distrust also needs to be taken seriously. Distrust can provide clues as to what is going wrong or what might be the points of contention between the public and their perception of what constitutes public benefit and that of the institutions involved. For example, the disquiet voiced by the public regarding private-public partnerships points to the expectation of the public that public institutions act in their interest and provide and promote public benefit. Citizens may fear that such partnerships may not distribute benefits equally: while there is value in proposing rigorous data governance frameworks for public-private partnerships, this might not address people’s concerns about whether the public, including vulnerable and marginalised groups, will appropriately benefit from large data collection and sharing programmes. Hence, despite efforts to avoid distrust through governance and engagement strategies, people might still not trust.

With public trust depending significantly on the commitment of public institutions to contribute to the public good, large-scale genome programmes need to be clear about expected societal benefits as well as possible risks. Whilst research has given useful insights into the role of genomic variation in many rare diseases, we also know that genomics plays a more limited role in the aetiology of most common diseases than in many rare diseases. This, in turn, means that the stratifying populations on the basis of genomic variation in common disease provides limited insight into [absolute] risks of disease. It has been argued that the promissory strength of genomic medicine lies not only in its potential to promote health but also science, innovation, and the industry of countries and nations [7, 8]. At the workshop, it was suggested that drivers for continuous investment in genomic initiatives, despite their limited demonstrated health benefit, might in some cases be motivated by the interest to collect more data for research (in the public and private sector), the need to secure funding for research projects, or to help promote the life sciences and data industries. Some participants also expressed the concern that focusing on genomics as the principal route to disease treatment and prevention for the majority of conditions and populations could intensify genetic or biological determinism and downplay the significance of social determinants for health [9]. This could cause the diversion of important resources to genomics and away from other effective approaches, including approaches that focus on non-medical factors that influence health, disease prevention and treatment.

The Genomics England Generation Study was discussed as an example of a genomics initiative that has the potential to lead to important societal benefits, while highlighting that it has yet to be determined what its benefits might turn out to be in practice. The Generation Study is a research programme recruiting through the English National Health Service and running in tandem with the national newborn screening programme [10]. Launched in 2024, it aims to screen 100,000 newborn babies for more than 200 genetic conditions and explore the potential for genome sequencing to improve health outcomes for newborns with rare genetic conditions. To date, most newborn bloodspot screening programmes rely mainly on finding very early markers of disease, where there is some evidence that this will reduce morbidity and mortality. Their uptake is generally high; indeed, they are mandatory in many countries [11, 12]. The aspiration is that whole genome screening will predict even earlier (than biochemical markers) which diseases will manifest and be able to do so at a reasonable cost, since many diseases can be tested for in one test. Therefore, the aim of the Generation Study is to generate evidence regarding the effectiveness and cost-effectiveness of whole-genome newborn screening.

The Generation Study was presented as an important study with the potential to yield interesting results, but several concerns were also raised. For example, in the workshop, it was acknowledged that many of the diseases tested for might be too rare to occur in this ‘pilot’ of 100,000 babies. The dual aim of the study—to conduct open-ended research whilst also providing care through screening and disease detection—was thought to raise issues around informed consent. Participants highlighted the importance of ensuring that parents properly understood the ‘hybrid’ nature of the study [13]. Some of the discussants also raised the issue of the cost to healthcare systems of babies with a genetic prediction who would require regular surveillance but do not go on to develop the condition. It was highlighted that our current understanding of the relationship between genetic variant and disease comes largely from the diagnostic setting. A variant does not act in isolation, but its expression (or not), i.e. its penetrance and expressivity, frequently relies on complex temporal, cellular and environmental factors [14]. Therefore, the ability of a genetic variant on its own to predict serious disease is likely to be quite low [15]. Finally, some of the workshop discussants suggested that any calculations of the effectiveness, including cost-effectiveness, of a genomic newborn screening study, such as the Generation Study, should take into account the potential impact on the availability of diagnosis, care and treatment for children who are already sick.

The debate that ensued from discussing the societal benefits of large-scale genomic programmes, such as might be generated in the context of the Generation Study, highlighted the difficulty of evaluating what should be counted as societal benefits in a context where boundaries between healthcare provision and research are blurred [16]. This being said, what all participants agreed upon is the importance of being aware not only of the potential benefits of such initiatives, but also of potential disadvantages and costs large genome screening programmes may hold, and to critically reflect on how to achieve the objective of public healthcare systems to provide health benefits to all. For this purpose, robust research is needed prior to rolling out any such programmes within the public health sector.

## Economic level

Economic evaluations of the costs and benefits of genomic technologies present a considerable challenge for health economists, policy-makers, clinicians and patients [17]. Though there have been some efforts to evaluate the cost-effectiveness of specific applications of genome and exome sequencing, the current health economic evidence-base data to support the widespread use of these technologies remains limited [18]. Moreover, evidence on the economic value of sequencing at the population level is in very short supply. One such case, though including only a small subgroup of the population, is the use of sequencing in a screening context, perhaps via a screening programme for high-risk cancers. A small number of model-based economic evaluations of such applications have been published that give a signal of potential cost-effectiveness, albeit with considerable uncertainty [19, 20]. A second case is to consider the economic value of large-scale sequencing programmes, which often have a research focus (e.g. the 100,000 Genomes Project in England focused on patients with rare disease or cancer). It is important to think about health economic value at this broader level because it may not necessarily be cost-effective to introduce a genomic medicine service, just because there are specific sequencing applications within that service that are cost-effective. Alternatively, it might be the case that specific applications are only cost-effective when offered as part of a national sequencing service, because a certain level of testing infrastructure is required. However, although some evidence exists on the potential for such programmes to avoid costly diagnostic odysseys [21], there are no studies evaluating the broader economic value of these large sequencing programmes. Moreover, the broad costs and consequences (positive and negative) of disclosing genetic risk information to individuals and using this information in clinical practice have not yet been adequately explored [22]. Questions remain as to what sort of (possibly life-long) clinical follow-up, (potentially invasive) clinical testing, and risk-management will be needed, and what level of (potentially inappropriate) medicalisation of healthy people, including children, will result.

At the workshop, the Genomics England Generation Study was discussed as providing an opportunity to advance understanding of economic value in this context via an independent multidisciplinary evaluation of the feasibility, clinical utility and cost-effectiveness of newborn genome sequencing [23]. While it was questioned whether the numbers of infants diagnosed with any one rare disease will be sufficient to develop robust estimates of costs and benefits, and whether the timeframe for the pilot will be adequate, it was agreed that the development of a formal value assessment framework for large population sequencing initiatives could be beneficial to also contribute to improving evidence on economic value in this space. The Economics Subcommittee of the International Consortium on Newborn Sequencing is bringing together health economists working in this clinical context to develop such a framework, and to coordinate the collection of outcomes data to better inform policy decisions [24].

It was further discussed that the way healthcare systems are structured, governed and financed can have an impact on the ability to evaluate how large-scale genomics programmes can best benefit society. For example, the Danish healthcare system is decentralised and organised at three levels: national, regional and local. This layout makes it difficult for Denmark to evaluate its National Genome Centre’s programme, as this separation contributes to difficulties in allocation of funding, as well as in accessing data when taking decisions on implementing evaluation programmes [25]. However, the root of the problem is not the split between regional and national level (though the split might of course slightly strengthen the real cause of the problem), but namely lack of resources or prioritising of resources for economic evaluation of the Danish programme for implementation of whole genome sequencing in healthcare. When the national large-scale programme was designed and funded, the focus was first and foremost on benefiting patients as soon as possible. Therefore, the selection of relevant patient groups that would be able to benefit from this programme, and the implementation of whole genome sequencing across the country within the selected patient groups was the priority. Unfortunately, no economic impact analyses were carried out and no resources were allocated to future economic evaluation or impact analyses of the national programme.

In order to carry out analyses of the economic impact of implementing whole genome sequencing in healthcare, a great deal of data is needed. But it is often difficult for clinicians to make time in their daily routine to identify the relevant data in patient records and provide them to national registries. Therefore, the relevant data is (currently) only accessible in patient records (even in Denmark, boasting one of most digitised healthcare systems in the world) yet not accessible at the Danish National Genome Center or other national registries. Hence, the Danish context demonstrates the negative impact of the absence of a relevant evaluation framework. Currently, evaluations mainly compare pricing or present micro-costing studies; the impact at the individual stakeholders’ clinical or societal level is not considered. More research is required in this field to broaden the value assessment framework, because the evaluation of the economic impact of whole genome sequencing, and linked health data is key to budget allocation at all levels including research, clinical trials and industrial competitiveness [26].

## Clinical level

To ascertain the societal value of large-scale genomics programmes, it is important to examine the stakeholders’ level benefits that might accrue in daily practice, for patients and their families, and also for healthcare professionals and systems. By looking at an individual’s genome, it may be possible to identify the genetic cause of, or contributions to, a health condition, or genetic variation that may increase the risk of an individual developing a disease later in life [27]. These insights could ultimately help healthcare professionals offer targeted actions to manage risk. For example, genomic testing might provide information about how a patient will respond to different medications and treatments or guide the type and frequency of surveillance. As yet, evidence to inform whether such data translates into positive outcomes, or about the impacts on individuals of learning about a genetic health risk remains limited [28]. An important emerging benefit of rare disease genetic diagnosis is that genetic therapies are now a realistic prospect for an increasing number of rare single-gene disorders [29]. Furthermore, knowledge about some types of genomic knowledge can guide family planning and cascade testing, allowing benefits for the broader family.

Genome sequencing is becoming part of routine clinical practice, e.g. in oncology or paediatrics for children with rare diseases. In England, the NHS Genomic Medicine Service was launched in 2018 to ensure equitable access to genetic and genomic testing across the country. Most patients (and their families) who receive a genetic diagnosis report positive experiences regarding clinical benefits such as targeted treatment [30], or practical or psychological benefits such as contact with support groups or relief from feelings of guilt [31, 32]. For some patients, however, receiving a diagnosis does not end their concerns and fears, and some feel frustrated and lose hope when receiving a diagnosis if there is no further information about their condition or no available treatment [33, 34]. Genome sequencing will not provide a clear diagnosis for all (e.g. only a quarter of patients selected on the basis of having a likely molecular diagnosis will receive one) [21]. While negative feelings like loss of hope, isolation and lack of specific support may result, it is also clear that development and research in genomics allows people to gain new hope, for example, that they might receive a result in the future [35]. For people who participate in national genomics programmes that combine research and clinical care, knowing that their— or their child’s—genomic data might be revisited when research has generated new knowledge, has been identified as a coping strategy for patients. Crucial to this reaction will be to properly manage these hopes and be careful about not raising false hopes with an overly positive genomic rhetoric.

A similar mechanism of seeing genomic medicine as an open-ended endeavour involving the possibility to achieve certainty at some point during the lifetime of a patient can be observed among professionals involved in next-generation sequencing to find genetic diagnostic answers to complex developmental, physical and neurological disorders. A multi-site study in a large Centre for Human Genetics in Belgium describes how laboratory professionals, clinical geneticists and other physicians navigate uncertainties when interpreting the vast amount of complex data that comes from genomic sequencing [36]. It demonstrates that many professionals acknowledge the uncertainties in their field, and at times even welcome them as an opportunity to use their ‘clinical intuition’ as a decisive element or to even relativise a negative result to give hope to patients. The uncertainty is used as a justification for accommodating various types of professional expertise, experience, interpretations, and also normative attitudes about the best pathway for a patient, while being able to offer a certain degree of optimism. Also, other studies show how uncertainty regarding a genetic result is used in clinical practice to keep the door open to hope, promise, and potentiality [37]. This turn to functional uncertainty gives professionals greater leeway in making decisions on what the best care should look like in a given case. However, the views and preferences of patients themselves do not always seem to be taken into account [38]. This raises questions about the centrality of the place of patients in ‘personalised’ medicine [36].

The examples above illustrate that genomic medicine can provide guidance relevant to everyday decisions for patients or physicians, but perhaps not the expected clear guidance that could prove helpful in navigating uncertainties and hopes in everyday practice. To avoid ambiguity in care decisions and the decrease of patients’ trust in medical expertise, it will be important to be transparent about the values and interests underlying the decisions that are made following a genomic result. Furthermore, it is important to openly address current limitations and temper the promises and enthusiasm of early diagnosis of disease and intervention through whole genome sequencing.

## Scientific level

When it comes to the value of genomics at the scientific level, our workshop chose to focus on the scope and limits of large-scale genomics screening used in the context of polygenic scores or precision cancer medicine from the perspective of current scientific advances.

The 2020 UK Genomic report presents polygenic risk scores (PRS) as a new generation of risk prediction tools to inform clinical decisions as well as screening policies [39]. PRS are calculated by examining the genetic contribution of multiple common genetic variants across the genome to determine the genetic contribution to common diseases such as cancer or coronary artery disease. However, the aetiology of most common diseases is multifactorial, with the total genetic component often being in the range of 20–40% [40]. PRS cannot say anything about the 60–80% of total risk that comes from other sources, such as environmental, socio-demographic, epigenetic or stochastic factors such as immune repertoire. Yet media coverage of PRS often pitches them as similar to rare disease genetics, hence the expectations they engender may not be possible to meet [40]. It has been suggested that screening programmes for certain cancers might be improved if those with high polygenic risk scores receive more or earlier screening, whilst those with lower scores might start their screening later. However, this may lead to false reassurance, such that those with a low PRS disengage from screening programmes. Conversely, PRS may bias towards overdiagnosis where the person does not benefit from its detection [40].

It was discussed in our workshop that when rolling out large genomic screening programmes, it is important to emphasise and acknowledge both the possibilities and the limits of the scientific discoveries when applied to the clinic or ‘real world’. For example, national genome sequencing programmes focus on precision oncology to improve diagnosis and target treatment. Remarkable achievements have been made through whole genome sequencing, enhancing the understanding of cancer, including risk assessment, early prevention and effective care. However, whole genome sequencing does not always provide straightforward responses and raises several questions regarding the interpretation and conclusiveness of findings in the case of a particular patient [41]. To reduce the uncertainty of results of genomic testing in the clinical context, it has been found that the involvement of multidisciplinary Advisory Boards or a systematic evaluation framework for clinical actionability can be very helpful in making sense of genomic findings [42]. The European Society for Medical Oncology (ESMO) has developed such a framework supporting the interpretation of variants in individual patients’ tumours and aims to avoid over-interpretation and treatment. Research has shown that these frameworks can help identify and prioritise appropriate therapies and improve survival duration and quality of life of patients with cancer [43]. Furthermore, as international collaborations between national genomic programmes grow, it has been suggested that the creation of a global network of multidisciplinary organisations could help make best use of learnings from these initiatives to further the adoption of genomics into cancer care [44]. The workshop participants agreed that continued rigorous research exploring the realistic scope as well as the limits of genomic programmes is needed to make them useful for the clinic.

## Population level

Finally, the workshop discussions turned to the question of what overall value genomic medicine holds for populations and public health programmes that take into account the interaction of genetics, lifestyle and the environment to advance prevention, diagnosis and treatment on a population level. Such programmes do not only deliver on the immediate needs of an individual patient but may also contribute to a database that can be used for insights to improve public health more broadly. Indeed, precision public health projects have the potential to improve delivering the right interventions to the right populations at the right time, at present and in the future [45].

While the use of genomics in public health, particularly in the context of pathogen genomics [46], is a promising field with many opportunities, it has been stated that effective and responsible translation of genome-based knowledge in the context of human genetics into larger public health programmes is currently a work in progress [47, 48]. Challenges to successfully implementing public health genomics include the tensions between precision and population-based strategies; i.e. it is important to recognise the difficulty in implementing individually tailored preventative or therapeutic interventions that reject a ‘one size fits all’ approach to a broad population level, which requires approaches for ‘all’. This requires the identification of complex multi-level risk factors and their impact on a population and the translation of this information to develop targeted public health programmes [47, 49]. However, as aforementioned, there is still a lack of an evidence-based evaluation showing that genomics improves public health outcomes and that all relevant multi-level risk factors are identified to successfully target public health programmes.

Indeed, it is important to remember that behavioural factors and social determinants of health may play a larger role in public health than genomic programmes might reveal [50]. And though genomics holds potential for improving health at the population-level, it remains the case that minority groups, rural communities and other marginalised groups are less likely to participate in and benefit from precision public health programmes [45]. Hence, health equity and the appropriate return of benefits of genomic medicine, especially among developing nations, are difficult to evaluate at this point.

Based on the example of the Plan France Médecine Génomique 2025, limits can be observed in obtaining and providing maximal value for the population at large [51]. While the Plan clearly emphasises the values of equity and justice, transparency, and democratic use of knowledge, at the same time, it underlines the importance of economic development, competitiveness and knowledge acceleration. This can create tension between attaining direct benefits to public health and the need for scientific and economic achievements. Unless this juxtaposition is appropriately balanced, it can be of detriment in providing benefits to the population, and lead to the observed over-promising around scientific and economic advances in genomics, placing these interests above the aim of providing equitable care and improving health for all members of society, and the health of marginalised groups in particular [47]. Important steps to counter such developments include, as already concluded in previous workshops, transparent, accountable, and clear communication of the interests of large-scale genomic programmes, and of the expected benefits, challenges and realistic outcomes for the public, i.e. the population at large [1, 4].

## Discussion

Our exploration of the values of large-scale genomics programmes has demonstrated the complexity of such an evaluation. At present, although there has been a plethora of studies on the outcomes and impact of genomic medicine, there has not been sufficient analysis of the benefits at various levels of societies, individual stakeholders, populations and so on. This is not to say that such an endeavour is impossible; one example in the area of collecting and governing data use can be found in the development of a digital Public Value Assessment Tool (PLUTO) to ensure a data-solidarity governance framework [52]. The latter consists of assessing how much public value the use of data for a specific purpose will likely generate insofar as benefits to the environment, economy, and society are concerned. At this point, PLUTO is still in its pilot phase, and it remains to be seen if it can adequately respond to the many complexities involved in assessing public value of data-driven healthcare technologies, and whether this would even be applicable to all cases, including the implementation of genomic medicine.

Despite the current lack of evidence on the economic value of large-scale genomic programmes, they are being pursued and financed without adequate upstream or downstream evaluation of their expected benefits alongside their associated costs. Yet, public resources are being allocated to these programmes, with the risk of defunding other health programmes that could bring about tangible benefits to society. More appropriate policies regarding resource allocation aimed at the maximisation of health benefits for all groups in society could result from a more robust and broader evaluation of benefits that takes into account the various levels at which benefits and costs might arise. These benefits and costs that are likely to emerge at each level should be considered together and balanced against each other.

Our workshop discussions have shown that in order to ensure that large-scale genomic programmes are beneficial to all, and thus warrant the public’s trust and support, various criteria need to be taken into account when planning their roll-out and implementation. These include:

- Avoiding overpromising on genomic outcomes. Stakeholders involved in communicating on the possibilities that large-scale genome sequencing offer must present a balanced and open discourse on the practical scope and limitations of genomic medicine.
- Generating evidence on economic value. To date, no studies have thoroughly evaluated the economic value of large-scale genomic sequencing programmes. Such studies are required to rigorously determine the health economic benefits of these large genomic programmes. These assessments should then guide healthcare resource allocation decisions for genomics programmes as they do for other health innovations that are implemented in the public health sector.
- Understanding the various and diverse patient, family, and physician experiences. Though genome sequencing holds a number of promises, it is not always able to provide clear-cut diagnoses or directions for specific care or treatment pathways for all patients. This leads to different reactions on the part of both medical personnel and patients. Navigating the expectations, hopes, uncertainties, and disappointments has emerged as a predominant concern to be addressed.
- Prioritising specific target groups when implementing genomic sequencing programmes. It is necessary to proceed cautiously and base every step of these programmes on evidence, beginning with more targeted groups before extending sequencing to the population at large.
- Better addressing the tensions between promises to improve individual health through personalised medicine, to promote the overall utility of public health objectives, and to drive scientific and economic achievements.
- Large-scale genomic sequencing programmes must actively seek to reduce rather than exacerbate existing health inequalities with underrepresented groups. To do this will require, amongst other activities, collaborating with community stakeholders to build trust and co-design research, ensuring consent processes are robust and sensitive to cultural contexts, and establishing mechanisms for communities to provide input and raise concerns.

## Conclusion

Our workshop highlighted that there are different kinds of benefits of genomics at various levels of society that should be taken into account when implementing large-scale genomics programmes. Despite the important insights regarding these benefits that have been gained at the workshop, we established that more in-depth analyses of the benefits and limits of genomics within each of the levels discussed are needed. Until these are accomplished, a translational path for the benefits of genomics to reach clinical practice remains incomplete and thus questions the return of benefits to all groups and communities of society. Not only must more studies be carried out, but also political decisions and the development of policy must take these studies into account. Alongside further analyses of values and benefits, the need for a renewed focus on the issue of equity in the realm of genomic medicine has emerged. This requires taking into full account communities deprived of equitable access to genomic medicine regarding a number of aspects: representative and diverse genetic data resources; access to adequate genomic testing/diagnoses and healthcare; continuous social security safety net during and after treatment; extended family care if needed; lifespan care and follow-up in tandem with new genomic discoveries. One step towards achieving these goals could involve reinforcing the articulation of Europe’s different national genomic programmes with the European Health Data Hub, as envisaged in the One Million Genome Initiative.
